# Gastric Polyps Detected Incidentally during Gastroscopy and Follow-Up Results

**DOI:** 10.3390/jcm13113117

**Published:** 2024-05-26

**Authors:** Mehmet Onur Gul, Selda Oguz Aslayan, Kadir Corbaci, Aytac Selman, Emre Berat Akcay, Zehra Unal Ozdemir, Hakan Ozdemir, Cebrail Akyuz

**Affiliations:** 1 Surgical Oncology Clinic, Malatya Training and Research Hospital, Malatya 44330, Turkey; 2 General Surgery Department, Üsküdar State Hospital, Istanbul 34662, Turkey; seldaaslayan@gmail.com; 3 General Surgery Department, Osmaneli Mustafa Selahattin Çetintaş State Hospital, Bilecik 11500, Turkey; dr.kadircorbaci@gmail.com; 4 General Surgery Department, Haydarpasa Numune Training and Research Hospital, Istanbul 34668, Turkey; aytacselman@gmail.com (A.S.); dr.eberatakcay@gmail.com (E.B.A.); drzehraunal@gmail.com (Z.U.O.); hakanzdmr@yahoo.com (H.O.); drcakyuz@hotmail.com (C.A.)

**Keywords:** colonic polyp, fundic gland polyp, gastric cancer, gastric polyp, hyperplastic polyp

## Abstract

(1) **Background:** We aimed to identify the possible relationship between various diseases of the upper digestive system and colon polyps by analyzing patients with gastric polyps and evaluating the cancers and diseases accompanying the polyps. (2) **Methods:** Each patient’s age; gender; polyp type and size; presence of *Helicobacter pylori* (*H. pylori*), atrophic gastritis, and intestinal metaplasia; status of whether cancer developed during follow-up; status of whether a colonoscopy was performed or not; and colon pathologies detected during colonoscopy were analyzed retrospectively using hospital records. (3) **Results:** Between the study dates, 19,214 esophagogastroduodenoscopies were performed in the endoscopy unit of our hospital. Gastric polyps were detected in 178 (0.9%) patients. No significant relationship was found between the gastric polyp size and the occurrence of gastric cancer or gastrointestinal system malignancy (*p* > 0.05). A colonoscopy was performed in 86 of the 178 patients who underwent gastroscopy. The frequency of polyp detection during colonoscopy was statistically significantly higher in patients with gastric polyps than in patients without gastric polyps (*p* < 0.001). (4) **Conclusions:** New prospective studies are needed regarding the relationship between gastric polyps and gastrointestinal system diseases. Going forward, a colonoscopy will be required in gastric polyp patients, especially with FGP.

## 1. Introduction

The epidemiology of gastritis, gastric atrophy, and gastric lesions associated with increased acid imbalance has undergone significant changes, and the frequency of polyps is thought to be increasing [1]. Over 90% of polyps are asymptomatic and may be found incidentally. Larger polyps can cause issues such as bleeding, anemia, abdominal pain, and stomach obstruction. Although typical appearances of some types of polyps can be seen using endoscopy, a histological evaluation is required to determine the presence of dysplasia. Therefore, a polypectomy should be performed for all gastric polyps to assess the pre-malignancy risk [2].

The three most common types of benign epithelial gastric polyps (BEGPs) are fundic gland polyps (FGPs), gastric hyperplastic polyps (GHPs), and adenomatous polyps. If more than one stomach polyp is present, they are usually of the same histological type [3]. Stomach polyps are classified as epithelial (fundic gland polyps, hyperplastic polyps, adenomatous polyps, hamartomatous polyps) and non-mucosal intramural polyps (gastrointestinal stromal tumors, leiomyoma, inflammatory fibrinoid polyps, fibroma, lipoma, ectopic pancreas, neurogenic tumors, and neuroendocrine tumors) [2].

Colorectal cancers are known to be the most common concurrent cancers in patients with stomach cancer [4]. Colorectal cancer is the third most common type of cancer in the world [5]. The relationship between stomach polyps and colon cancer is an important one due to its frequency. A study conducted in 2013 showed that the presence of concurrent colonic neoplasias increased in patients with gastric adenoma or cancer, and a pre-treatment screening colonoscopy is recommended [6]. One study in the literature showed that *Helicobacter pylori* (*H. pylori*) infection increases the risks of colorectal adenoma and nonadenomatous polyps in patients under the age of 50 with gastric polyps and low-grade intestinal neoplasia [7].

Many studies still cannot provide clear information about whether gastric polyps increase the incidence rate of cancer. To solve this problem, we aimed to identify the possible relationship between various diseases of the upper digestive system and colon polyps by analyzing patients with gastric polyps and evaluating the cancers and diseases accompanying polyps.

## 2. Materials and Methods

This study was evaluated by correlating the follow-up periods of gastric polyps and the presence of cancer with gastrointestinal system diseases that occurred during this period. This retrospective study group consisted of patients diagnosed with gastric polyps during gastroscopy in the endoscopy units of the University of Health Sciences Haydarpaşa Numune Training and Research Hospital between January 2016 and May 2021. The patients were selected based on their endoscopic examination results and histopathological findings.

The demographic characteristics, polyp characteristics (size, number, location), endoscopic images, histopathological results, and clinical follow-up periods of the patients were collected from hospital records. Patients diagnosed as have polyps during gastroscopy whose pathology results did not indicate the polyp type were excluded from the study. In our study, no polyp patients were diagnosed with gastrointestinal stromal tumors, considered a nonepithelial polyp type. Twenty-one patients who were detected as having polypoid lesions during gastroscopy, followed up, and found to have foveolar dysplasia due to their histopathology were excluded from the study. One patient’s gastric polyp pathology revealed foveolar hyperplasia and a hyperplastic polyp was detected during the follow-up endoscopy polypectomy. This patient was included in the hyperplastic polyps group. Likewise, four patients with xanthoma as a result of their histopathology were excluded from the study. Patients diagnosed with polyps during their gastroscopy and chronic active or inactive gastritis after their histopathological evaluation and who had the same results during their follow-up period, as well as patients who could not be followed up, were excluded from the study. Each patient’s age; gender; polyp type and size; presence of Helicobacter pylori (*H. pylori*), atrophic gastritis, or intestinal metaplasia; status of whether their cancer developed during follow-up; status of whether a colonoscopy was performed or not; and colonic pathologies detected during colonoscopy were analyzed retrospectively. The results for the patients who had a follow-up endoscopy were analyzed. The gastric polyp types and the presence of colon polyps were evaluated statistically. In our study, the polyp dimensions in the histopathological evaluation were considered for calculation rather than the polyp size in the endoscopy report. This study aimed to examine the rates of malignancy development in the follow-up period of gastric polyp patients who were not diagnosed with malignancy. For this reason, patients with gastric adenocarcinoma and neuroendocrine cancer seen on their first endoscopy were excluded from the study.

Statistical analyses were performed using IBM SPSS software, version 23.0 (IBM Corporation, Armonk, NY, USA). The average age, gender, and clinical findings of the individuals included in the study were determined using descriptive statistical methods and their frequencies and percentages. We examined whether the numerical variables showed a normal distribution using visual and analytical methods. The chi-square and Fisher’s exact tests were used for the categorical and nominal variables in pairwise comparisons. In all analyses, *p* < 0.05 was considered statistically significant.

## 3. Results

**Figure 1:**
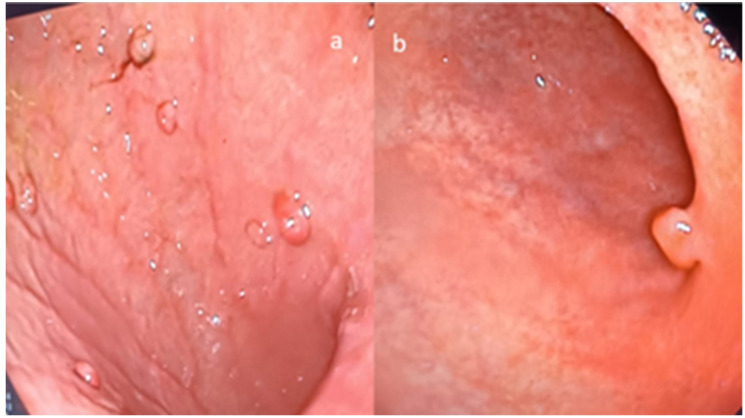
Endoscopic image of polyp types: (**a**) multiple-location fundic gland polyp; (**b**) hyperplastic polyp appearance.

Between the study dates, 19,214 esophagogastroduodenoscopies were performed in the endoscopy unit of our hospital. Gastric polyps were detected in 178 (0.9%) patients (Figure 1). The demographic data and polyp types, sizes, and locations are given in Table 1. Gastric cancer developed during follow-up in 3 patients with gastric polyps. The average time until the follow-up of these three patients, in whom gastric cancer was detected during the follow-up endoscopy, was 71.3 months, with a median of 81 (range 36–97) months. In the first endoscopy results from the patients with gastric cancer, it was observed that two patients had hyperplastic polyps and one patient had an adenoma. The first of the 3 gastric cancer cases was a 62-year-old female patient. In her first gastroscopy, a polypectomy was performed for a 1 cm polyp in the antrum, and as a result of the histopathological examination, it was found to be a hyperplastic polyp. During the gastroscopy performed in the 29th month of the follow-up period due to increased abdominal pain, the biopsy taken from the hard ulcerated area covering the greater curvature was reported as signet ring cell carcinoma. The patient was diagnosed with peritoneal carcinomatosis based on computed tomography results performed for staging, and the treatment was initiated by the oncology specialist. Exitus occurred due to a massive pulmonary embolism in the 7th month of treatment. Another cancer developed at the age of 80, after which a gastric polyp in the cardia was diagnosed and a polypectomy was performed. As a result of the histopathological evaluation, it was found to be a hyperplastic polyp. In the control gastroscopy performed after 92 months due to the patient’s complaints of weight loss and abdominal pain, a mass lesion was observed surrounding the lumen, starting from the cardia and extending to the distal part of the corpus. The pathology result was reported as adenocarcinoma. The patient had peritoneal carcinomatous findings in their radiological imaging results and could not be operated on, could not receive oncological medical treatment, and was given palliative treatment. The patient developed exitus 4 months later. A 44-year-old female patient, who underwent gastroscopy due to treatment-resistant gastroesophageal reflux, underwent a polypectomy for a 1.5 cm polyp in the corpus. The histopathological evaluation result revealed it to be an adenomatous polyp. No pathology was found in the patient’s first-year follow-up gastroscopy results. Approximately 33 months after the first gastroscopy, a mass lesion involving the cardia and corpus was observed in the follow-up gastroscopy performed due to the recurrence of abdominal pain and reflux complaints, and a biopsy was taken. Since the biopsy revealed adenocarcinoma, the patient underwent a total gastrectomy. The patient’s sample pathology was T4N1M0. The patient, who received chemotherapy after the operation, was disease-free in the 48th month. No significant relationship was found between the gastric polyp (GP) size and the occurrence of gastric cancer or gastrointestinal system malignancy (*p* > 0.05).

During the gastroscopy, the number of patients positive for *H. pylori* was 31 (17.4%), while the negative patients equaled 147 (82.6%). The atrophic gastritis result was positive in 13 (7.3%) patients and the intestinal metaplasia result was positive in 44 (24.7%) patients out of 178 total patients. The GIS malignancy rates of patients with positive *H. pylori* results were higher than those with negative ones, although the result was not statistically significant (*p*: 0.053). The relationship between *H. pylori* and the GP type is shown in Table 2. The rate of *H. pylori* was 26.7% in hyperplastic polyps and 25% in NET (Table 2). No statistically significant differences were found in terms of the frequency rates of GIS malignancy and gastric cancer based on the GP types (Table 3). It was observed that of the patients with GIS malignancies that developed during follow-up, three (25%) were gastric adenocarcinoma, three (25%) were colon adenocarcinoma, three (25%) were hepatocellular cancers, two (16.6%) were gallbladder tumors, and one (8.33%) was a cholangiocellular cancer.

A colonoscopy was performed in 86 of the 178 patients who underwent a gastroscopy with GPs. Colon polyps were detected in 32 patients and colon TMs were detected in 3 patients during the follow-up. During the colonoscopy, 4 (12.1%) cases were detected in the cecum, 2 (6.1%) in the ascending colon, 2 (6.1%) in the transverse colon, 4 (12.1%) in the descending colon, and 11 (33%) in the sigmoid colon, while 3 patients (9.1%) had cases in the rectum and 7 patients (21.2%) had cases with multiple locations. 

The relationship between the gastric polyp type, patients with polyps detected during colonoscopy, and polyp subtypes is given in Table 4. The type of polyp in the stomach of three patients with colon cancer was found to be a hyperplastic polyp. Familial adenomatous polyposis (FAP) was detected in one of seven patients with multiple polyps in the colon. The patient’s operational and follow-up findings could not be accessed because the follow-up was carried out in another center. The numbers of polyps in the colonoscopy results of the remaining six patients were less than 10, and attenuated FAP or FAP was not considered. The patients underwent a polypectomy and no colon cancer developed during the follow-up. Patients with fundic gland polyps and hyperplastic polyps were compared statistically in terms of the frequency of colon polyps, and colon polyps were found to be statistically non significant with Bonferroni correction (*p* = 0.126). To compare the rate of colon polyps in patients with gastric polyps with the control group, colon polyp was detected in 4327 of 21,458 patients who underwent colonoscopy in the same period and did not have gastric polyps. They were evaluated by comparing them with each other. Colon polyps were detected in 37.2% of patients with gastric polyps who underwent colonoscopy. The polyp detection rate during colonoscopy in patients without gastric polyps was 20.2%. The frequency of polyp detection during colonoscopy was statistically significantly higher in patients with gastric polyps than in patients without gastric polyps (*p* < 0.001). The frequency of polyp detection during colonoscopy was 31.3% in patients with hyperplastic gastric polyps, 57.1% in patients with fundic gland polyps, and 20.2% in patients without gastric polyps. The difference between the three groups was statistically significant (*p* < 0.001). Post hoc pairwise comparisons (with Bonferroni correction) showed that the frequency of polyp detection on colonoscopy was statistically significantly higher in patients with fundic gland polyps than in those without gastric polyps (*p* < 0.001). Post hoc pairwise comparisons (with Bonferroni correction) showed that the frequency of polyp detection in colonoscopy was not statistically significant in those with hyperplastic polyps compared to those without gastric polyps (*p* = 0.168).

Follow-up gastroscopy was performed in 74 patients, and a repeat polypectomy was performed in 44. The median follow-up endoscopy duration was 15.5 months (1–122 months). NET type 1 was detected in 12 patients, and an average of 2.25 (+1.2) follow-up endoscopy procedures were performed on the patients. Since endoscopic submucosal or mucosal resection procedures are not performed in our hospital, these patients underwent polypectomy with follow-up esophagogastroduodenoscopy and were subsequently followed up with. There were two patients with hamartomatous polyps; one had a colonoscopy and no pathology, and one could not have a colonoscopy and follow-up testing in the long term. These findings were given considering that the pathologies that cause polyp formation in the gastrointestinal system will also cause other gastrointestinal diseases.

## 4. Discussion

Hyperplastic and fundus gland polyps account for approximately 90% of gastric polyps, followed by adenomas and other histological types, which are much less common. Although some studies state the opposite, Sonnenberg et al. reported the occurrence rate of hyperplastic polyps at 1.79% and fundus gland polyps at 7.72% [1,3,8]. Carmack et al. found that HPs and FGPs were the most common polyp types, accounting for 77% of all polyps [3]. In our study, GHPs and FGPs constituted 83.7% of all gastric polyps.

Fundic gland polyps (FGPs) constitute 8.3–72% of benign epithelial gastric polyps (BEGPs) and can be seen in approximately 8% of endoscopies [8,9]. They are usually 1–5 mm in diameter, with numerous transparent sessiles. These polyps are usually found in the corpus and fundus. FGPs are not associated with atrophic gastritis, and the prevalence rate of *H. pylori* infection in these patients is very low [10]. It has been reported that these polyps may disappear over time. Kapizoni et al. emphasized in their study that there is no clear evidence that PPI use increases the frequency of FGPs [11]. In addition, retrospective studies show that the average time for the development of FGPs is 32.5 months and that this regresses within three months after PPI discontinuation [12]. Dysplasia is seen in <1% of sporadic FGPs [13]. In a study with a large number of patients, the age of patients with incidentally detected FGPs was higher than for FGPs seen in patients with FAPs, and dysplasia did not develop in the incidental FGPs. The incidence rate was also lower [14]. The presence of dysplastic foci in patients with FGP should raise suspicion for FAP [15]. The risk of gastric cancer may be higher in patients with FAP [16]. A recent study has shown that patients with FAP have a higher incidence rate of gastric cancer [17]. The results of a comprehensive study by Genta et al. revealed that there is no significant correlation between the occurrence of sporadic FGPs and gastrointestinal malignancies. Therefore, they concluded that the occurrence of FGPs was not a reliable indicator of the development of gastrointestinal malignancies [10]. Some comments suggest that such an association with gastric cancer is due to the significantly lower prevalence rate of *H. pylori* in people with FGPs. There is a strong negative correlation between *H. pylori* infection and sporadic FGPs. Moreover, FGPs are rare in individuals infected with *H. pylori* [18]. It has been reported that colonic adenomas (7–45%) and adenocarcinomas (0–4.7%) may develop together or separately in patients with sporadic FGPs [19,20]. Additionally, Amarapurkar et al. reported that since the occurrence of FGPs increases the incidence of gastric carcinoma, the removal of gastric polyps via a polypectomy during endoscopy will prevent the formation of gastric cancer [21]. In our study, the lower incidence rate of *H. pylori* infection in FGPs compared to GHPs and other polyp types was statistically significant and consistent with the literature. During the follow-up period for patients with FGPs, no patient developed gastric adenocarcinoma. However, in line with the literature, the occurrence rate of colon polyps in patients who underwent colonoscopy while suffering from FGPs was statistically significant according to control group (*p* < 0.001).

Hyperplastic polyps (GHPs) constitute 28% of all benign epithelial gastric polyps and are sessile or pedunculated polyps measuring less than 2 cm in diameter [8]. Up to 80% of GHPs have been found to regress after *H. pylori* eradication [11]. In the study by Forte et al., it was found that GHPs recurred more frequently in the antrum than in the corpus localization [22]. A study reported that hypergastrinemia could be a risk factor in the malignant progression of hyperplastic polyps [23]. The prevalence rates of dysplasia in sporadic hyperplastic polyps have been reported to range from 1.9% to 19%, and this rate has a significant relationship with the size [24,25]. Up to 2.1% of resected polyps can develop into adenocarcinomas from hyperplastic polyps [26], with the risk being related to size. This observation has also found its way into the clinical guidelines, with recommendations to remove polyps that are larger than 10 mm [24]. In addition to GHP cancer transformation, cancer development from the surrounding mucosa has also been reported in patients with GHPs. Patients with GHPs require multiple biopsies of the surrounding mucosa, as the risk of adenocarcinoma in the mucosa is higher than in the polyp itself. Therefore, it is necessary to take multiple biopsies of the intervening mucosa to ensure proper diagnosis and treatment. In the study by Bar et al., the malignant transformation rate for GHPs was 1.9%, which was lower than the literature [27]. Since the etiology of relapse is still unclear, long-term follow-up seems necessary to detect and treat relapses to prevent poor outcomes in cancer transformations. However, a neoplastic component in 10% of GHPs does not support a watchful waiting strategy for all cases [22]. Most neoplastic polyps involve low-grade dysplasia, and high-grade dysplasia or early adenocarcinomas occur in only 1.6% (<10%) of cases. It is important to note that 8.4% of patients in the cohort showed GHPs measuring less than 10 mm. Intestinal metaplasia has emerged as a risk factor for the neoplastic transformation of GHPs, as shown in previous studies [22]. In our study, 2 out of 3 patients who developed stomach cancer had GHPs and 9 out of 12 patients who developed GI cancer also had GHPs. Although it was not statistically significant, it was observed that the cancer rate increased in the presence of GHPs.

Gastric adenomas are types of tumors that can lead to gastric cancer. They can be divided into three categories based on their structure: tubular, villous, and tubulovillous. They are relatively rare, making up only 0.69% of benign epithelial gastric polyps (BEGPs). While they can appear anywhere in the stomach, they are most commonly found in the antrum and tend to be solitary [3]. They often occur in the background of atrophic gastritis and intestinal metaplasia, although there is no proven relationship with *H. pylori* infection [3,28]. In our study, one patient had gastric adenocarcinoma that developed based on an adenoma. Colonic polyps were detected in 2 of the colonoscopies performed in this group.

Feng et al. found that the presence of stomach disorders such as GPs, *H. pylori* infection and atrophic gastritis increased the risk of colorectal polyps (CPs) (*p* < 0.001). This relationship was found to be significant in both univariate and multivariate analyses [28]. The same study also stated that colorectal polyps were statistically more common in hyperplastic and fundic gland polyps [28]. There are studies showing that the risk of CP increases in individuals with hyperplastic polyps and fundus gland polyps [29,30]. The mechanisms involved are still unclear. Gastric fundus gland polyps are the main components of gastric polyps, most of which are manifestations of long-term proton pump inhibitor treatments [3]. Reporting the opposite of this situation, Loke et al. reported that the incidence of gastric polyps in the groups of patients over 50 years of age with and without colorectal polyps was found to be statistically insignificant [31]. Therefore, some studies suggest that the etiology of positive relationships may be related to the decrease in the stomach acid barrier [3]. Although statistics cannot be produced by excluding all gastroscopy and colonoscopy results, it was thought that the colon polyp rates were proportionally higher in GP especially with FGP in our study. We believe that colon polyps in these patients are considered a precursor of cancer and that these results will lead to more meticulous colonic investigations by GPs with longer follow-up periods.

Inflammatory gastric polyps are complex solitary polyps, which are usually detected incidentally and covered with intact mucosa, often seen in the antrum and pylorus region [32]. Although they are often asymptomatic, they can cause severe findings such as gastric outlet obstructions and massive upper GI bleeding [33]. Gastric NETs are divided into four types, and type 1 NETs constitute 80–90% of all gastric NETs. Hypergastrinemia and *H. pylori* infection based on chronic atrophic gastritis are factors in the pathogenesis. The prognosis is excellent and the 5-year survival rate is over 95%. The presence of gastric intestinal metaplasia increases the risk of stomach cancer by six times [34]. The presence of IM under endoscopy is the most reliable indicator of atrophy. Patients with advanced gastritis, atrophy, and IM affecting both the antral and corpus mucosa should be considered at high risk for gastric cancer [35]. Gastric adenocarcinoma did not develop in any patient in this group, which is rarer than GHPs and FGPs and is thought not to increase the frequency rate of gastric cancer. Only two patients with inflammatory gastric polyps were accompanied by GIS cancer.

A limitation of the study is that it was not a prospective study and that a single person did not perform the endoscopy procedures. Failure to record the first GP location always means one cannot know whether the cancers and dysplasias that will occur are in the same or different locations. Another limitation of our study is that a colonoscopy was not performed on all GHP and FGP patients. Studies in which all patient groups can be evaluated homogeneously are required. Due to personal decisions and the lack of guidelines for gastric polyps, the physician responsible for the patient often changes the follow-up period depending on their own preference, and it is not known whether standardized work can be conducted thoroughly. The limitation of our study is that not all patients had 5-year and 10-year follow-up periods. For this reason, a study will be planned to evaluate the possible relationship between stomach and gastrointestinal cancers with long-term follow-up periods, including the same population as in our study. The limitation of our study is that the etiology of gastric cancer cases developing in GP patients cannot be fully elucidated and no comparison can be made by creating a control group.

The occurrence of *H. pylori* was found to be negative at a higher rate in FGP tests. Going forward, colonoscopy will be required in GP patients, especially with FGP. We state the necessity of examining the entire GIS in GP patients. New prospective studies are needed to standardize the treatment and follow-up protocols for all GPs.

## Figures and Tables

**Table 1:** Demographic findings and gastric polyp types.

	Mean ± SS/Number (%)	Minimum–Maximum
Age (year)	57.67 ± 14.33	18–88
Polyp Size (mm)	7.71 ± 3.92	2–58
Gender		
Female	110 (61.8%)	
Male	68 (38.2%)	
Polyp Types		Age Mean (+SD)
Hyperplastic Polyp	90 (50.6%)	60.3 ± 13.2
Fundic Gland Polyp	59 (33.1%)	52.7 ± 15.4
Inflammatory Polyp	9 (5.1%)	64.5 ± 10.8
NET ^1^	12 (6.7%)	53.6 ± 13.8
Hamartomatous Polyp	2 (1.1%)	47 ± 14.1
Adenomatous Polyp	6 (3.4%)	67.16 ± 6.46
Gastric Polyp Location		
Cardia	26 (14.6%)	
Fundus	22 (12.4%)	
Corpus	53 (29.8%)	
Antrum	43 (24.2%)	
Fundus + Corpus	13 (7.3%)	
Cardia + Fundus	3 (1.3%)	
Corpus + Antrum	14 (7.9%)	
Cardia + Antrum	4 (2.2%)	

^1^ Neuroendocrine tumors.

**Table 2:** Relationship between polyp types and *H. pylori*.

Gastric Polyp Type	*H. pylori* Positive (*N* = 31)	*H. pylori* Negative (*N* = 147)	*p*
Hyperplastic Polyp	24 (26.7%)	66 (73.3%)	0.006 *
Fundic Gland Polyp	2 (3.4%)	57 (96.6%)	
Inflammatory + Hamartomatous Polyp	1 (9%)	9 (90%)	
NET ^1^	3 (25%)	9 (75%)	
Adenomatous Polyp	1 (16.7%)	5 (83.3%)	
GIS ^2^ Malignancy			
Yes	5 (38.5%)	8 (61.5%)	0.053 **
None	26 (15.8%)	139 (84.2%)	

* Chi-square test. ** Fisher’s exact test. ^1^ Neuroendocrine tumors. ^2^ GIS—gastrointestinal system.

**Table 3:** Relationship between polyp types and malignancy and follow-up period.

Gastric Polyp Type	GIS ^1^ Malignancy		*p*	Gastric Cancer		Follow-Up Time (Months)
	Yes	None	Value	Yes	None	
Hyperplastic Polyp	9 (10%)	81 (90%)	0.080 *	2 (2.2%)	88 (97.8%)	71 (0–149)
Fundic Gland Polyp	0 (0%)	59 (100%)		0 (0%)	59 (100%)	65 (0–139)
Inflammatory + Hamartomatous Polyp	2 (18.2%)	9 (81.8%)		0 (0%)	11 (100%)	20 (1–140)
NET ^2^	0 (0%)	12 (100%)		0 (0%)	12 (100%)	62 (0–85)
Adenomatous Polyp	1 (16.7%)	5 (83.3%)		1 (16.7%)	5 (83.3%)	73.5 (36–146)

* Chi-square test. ^1^ GIS—gastrointestinal system. ^2^ Neuroendocrine tumors.

**Table 4:** Distribution and comparison of patients with gastric polyps and patients with polyps detected as a result of a colonoscopy.

Gastric Polyp Type	Colon Polyps		*p* Value	Colon Low-Grade Dysplasia	Colon High-Grade Dysplasia	Hyperplastic Colon Polyps
	Yes	None				
Hyperplastic Polyp	15 (31.2%)	33 (68.8%)	0.126 *	12 (25%)	1 (2.1%)	2 (4.2%)
Fundic Gland Polyp	12 (57.1%)	9 (42.9%)		10 (47.6%)	0 (0%)	2 (9.5%)
Inflammatory + Hamartomatous Polyp	2 (28.6%)	5 (71.4%)		1 (14.3%)	0 (0%)	1 (14.3%)
NET ^1^	1 (16.7%)	5 (83.3%)		0 (0%)	0 (0%)	1 (16.7%)
Adenomatous Polyp	2 (50%)	2 (50%)		1 (25%)	1 (25%)	0 (0%)

* Chi-square test. ^1^ Neuroendocrine tumors.

## Data Availability

The data from this study are unavailable due to privacy or ethical restrictions, meaning a statement is still required. However, we have data to be shared with the journal editor if the journal requests.
